# Adverse Events and Patient Outcomes Among Hospitalized Children Cared for by General Pediatricians vs Hospitalists

**DOI:** 10.1001/jamanetworkopen.2018.5658

**Published:** 2018-12-07

**Authors:** Mariam Krikorian Atkinson, Mark A. Schuster, Jeremy Y. Feng, Temilola Akinola, Kathryn L. Clark, Benjamin D. Sommers

**Affiliations:** 1 Department of Health Policy and Management, Harvard T.H. Chan School of Public Health, Harvard University, Boston, Massachusetts; 2 Kaiser Permanente School of Medicine, Pasadena, California; 3 Division of General Pediatrics, Boston Children’s Hospital, Boston, Massachusetts; 4 Department of Medicine, Massachusetts General Hospital/Harvard Medical School, Boston; 5 Department of Radiology, Lahey Hospital and Medical Center, Burlington, Massachusetts; 6 Division of General Medicine & Primary Care, Brigham and Women’s Hospital/Harvard Medical School, Boston, Massachusetts

## Abstract

**Importance:**

Pediatric hospital medicine is a relatively new and growing specialty. However, research remains inconclusive on outcomes for inpatients cared for by pediatric hospitalists compared with those cared for by general pediatricians.

**Objective:**

To analyze outcomes, adverse events (AEs), and types of AEs associated with care provided for pediatric patients by hospitalists vs general pediatricians.

**Design, Setting, and Participants:**

This cross-sectional study used data from the medical records of a US urban academic children’s hospital comprising 1423 hospitalizations between January 1, 2009, and August 31, 2015, for 57 diagnoses of patients cared for by either a hospitalist or general pediatrician. General pediatricians worked primarily in the hospital’s outpatient clinic, serving a few inpatient weeks per year, and were not the patients’ primary care physician. Data analysis was performed from July 1, 2017, to October 10, 2018.

**Main Outcomes and Measures:**

Outcomes were length of stay, total costs, 30-day readmission rates, and AEs. Adverse events were documented by *International Classification of Diseases, Ninth Revision, Clinical Modification* codes determined by review of medical records. Adverse event categories were drug events, infections, and device-related AEs. Generalized linear models were used to analyze patient outcomes, with standard errors clustered by physician. Models were adjusted for patient characteristics, including Chronic Condition Indicators. Models were estimated with and without adjustment for physician characteristics.

**Results:**

The data set contained 1423 hospitalizations among 726 female patients and 697 male patients (mean [SD] age, 6.1 [6.3] years). Hospitalists cared for 870 patients, and general pediatricians cared for 553 patients. Among the physicians, there were 57 women and 38 men; physicians were a mean (SD) 11.1 (8.1) years out of medical school. Patients cared for by general pediatricians were younger than those cared for by hospitalists (mean [SD] age, 5.4 [6.0] vs 6.5 [6.4] years; *P* = .001) but had similar mean (SD) Chronic Condition Indicator scores (1.5 [1.0] vs 1.5 [1.0]). A total of 33 of 56 general pediatricians (58.9%) and 24 of 39 hospitalists (61.5%) were women (*P* = .006), and general pediatricians were in practice twice as long as hospitalists on average (mean [SD], 16.0 [10.3] vs 7.9 [3.8] years out of medical school; *P* < .001). In multivariate models adjusting for patient-level features, there were no significant differences between general pediatricians and hospitalists for mean length of stay (4.7 vs 4.6 days), total costs ($14 490 vs $15 200), and estimated 30-day readmission rate (8.9% vs 6.4%), and results were similar with adjustments for physician characteristics. Device-related AEs were higher among hospitalists (3.0% vs 1.1%; odds ratio, 0.34; 95% CI, 0.12-1.00); this association became nonsignificant after adjusting for physician experience.

**Conclusions and Relevance:**

General pediatrician and hospitalist inpatient care had similar length of stay, total costs, and readmission rates. However, AEs differed between hospitalists and general pediatricians, with device-related AEs more common among hospitalists, which may be associated with hospitalists’ fewer years in practice. Such findings can inform hospitals in planning their inpatient staffing and patient safety oversight.

## Key Points

**Question:**

How do patient outcomes, including costs of care, readmissions, and adverse events, vary for inpatient care provided by hospitalists and general pediatricians?

**Findings:**

In this cross-sectional study analyzing nearly 6 years of medical records at a large US academic children’s hospital, patients cared for by hospitalists and general pediatricians had similar length of stay, total costs, and readmission rates. However, hospitalist care was associated with more adverse events related to medical devices.

**Meaning:**

As inpatient care increasingly moves away from general pediatricians toward hospitalists, this study suggests that hospitalist care is associated with more adverse events related to medical devices, suggesting an area of monitoring for future work.

## Teaser

This cross-sectional study analyzes length of stay, costs, readmission rates, and adverse events associated with care provided for US pediatric patients by hospitalists vs general pediatricians.

## Introduction

Since the 1990s, pediatric hospitalists have proliferated throughout the United States, and in 2016, pediatric hospital medicine became an official specialty recognized by the American Board of Medical Specialties. This recognition is believed to foster continued and much-needed efforts to standardize pediatric hospitalists’ functional roles relative to other types of physicians^1,2^ and reflects the larger evolution in outpatient and inpatient pediatric medicine. As medical advances have enabled greater management of chronic illness on an outpatient basis and as primary care has taken on more responsibility for addressing patients’ social complexity (ie, the psychosocial factors affecting patients), many community-based general pediatricians report difficulty interrupting their outpatient practice to perform rounds for hospitalized patients.^3^ Inpatient care has also grown more complex, which is associated with higher-acuity conditions presented by hospitalized children than previously.^4^

Owing to the growing prevalence of pediatric hospitalists, it has become increasingly critical to assess the effectiveness and outcomes associated with their care.^5^ Mixed evidence exists on whether hospitalists compared with general pediatricians provide care that enhances patient outcomes. For example, a 2006 review article found that patients of pediatric hospitalists have lower costs and 10% shorter lengths of stay (LOS) compared with traditional models of care in which primary care physicians perform rounds for their patients.^6^ However, another review of 11 studies focused on pediatric patients between 2 and 11 years of age concluded that the effects of the hospitalist model on quality and cost outcomes were heterogeneous, with varying findings on LOS, readmission rates, and patient satisfaction.^7^ Similar results were found in a systematic review of outcomes and quality measures in adult patients showing that, compared with nonhospitalists, hospitalists demonstrated greater efficiencies of care with the reduction of LOS and cost per stay, with mixed improvement in quality markers.^8^

Although published research continues to examine the comparison of hospitalists vs generalists for adult patients, to our knowledge, few recent studies have directly compared the outcomes of pediatric inpatients when cared for by hospitalists vs general pediatricians. This gap in research overlooks the recent trend in which general pediatricians are now increasingly focused on outpatient care.^9^ As general pediatricians’ provision of care increasingly shifts away from the inpatient context, questions emerge concerning whether general pediatricians have the experience to deal with the evolving and complex aspects of providing inpatient care, especially compared with hospitalists. A related area of interest is the prevalence of adverse events (AEs) and factors associated with AEs among hospitalized patients. Findings from studies that have examined the association between AEs and hospitalist care are inconclusive both in terms of the frequency and types of AEs.^10,11,12,13^ Understanding pediatric AEs in the context of hospitalists and other physicians therefore remains an important area for study.^14^

This research compares the outcomes of patients cared for by pediatric hospitalists and by general pediatricians in terms of LOS, total costs, 30-day readmission rates, and AEs. Drawing on approaches used in studies of adult care, we analyze these outcomes with and without adjustment for physician characteristics (eg, experience level and sex).^15^ Our analysis uses 6 years of detailed medical record data from a large, urban, US academic pediatric children’s hospital.

## Methods

### Data, Setting, and Study Population

We analyzed medical record data of hospitalized patients younger than 23 years who were discharged between January 1, 2009, and August 31, 2015, from an urban academic children’s hospital in California. The hospital provides care to young adults up to 25 years of age. However, only 6.3% of the hospitalizations in our sample (90 of 1423) were for patients older than 18 years, and our results are generally similar if we exclude these patients from the analysis. We included all patients with qualifying diagnoses across 7 subspecialty departments: cardiology, endocrinology, gastroenterology, hematology-oncology, neurology, pulmonology, and rheumatology. Patient data were deidentified, and the Harvard T.H. Chan School of Public Health Institutional Review Board approved the research as exempt. This study followed the Strengthening the Reporting of Observational Studies in Epidemiology (STROBE)^16^ reporting guidelines for cross-sectional studies.

We conducted interviews regarding physician assignment with the division heads of hospital medicine and general pediatrics who indicated that, during the period of our analysis, the physician assignment process was intended to be as random as possible. Patients were to be assigned to either service based on bed availability and census. However, there is still the possibility of some residual selection bias in physician assignment practices at the hospital. For instance, some patients could be admitted from outside emergency departments or transferred from other inpatient units, which could influence assignment to either type of physician. Differential assignments made by other services within the hospital were also possible. For this reason, we excluded surgical patients and those who were admitted directly into the intensive care unit.

The data for our analysis were obtained based on a larger study examining physician assignment practices at the study institution.^17^ For that parent study, the diagnoses in the sample reflected conditions within each subspecialty for which assignment may vary across generalists vs nongeneralists (see eTable 1 in the Supplement for a full list), which limited the inclusion of many high-volume and common patient conditions routinely cared for by generalists or hospitalists in the inpatient setting. We excluded subspecialist primary attending assignments, focusing solely on a comparison of hospitalists and general pediatricians reflecting a total of 57 principal diagnoses across the 7 subspecialties. Fifteen of 1438 hospitalizations in our data set were for patients initially admitted into the intensive care unit; they were excluded from the analysis, leaving a sample of 1423 hospitalizations.

### Outcome Measures

Patient outcomes focused on quality (readmissions and AEs), health care use (LOS), and total costs. We also examined types of AEs associated with hospitalist and general pediatrician assignments.

Length of stay refers to the total number of days a patient was hospitalized at the study hospital for a given encounter. Total costs were calculated based on total hospital charges multiplied by the cost-to-charge ratio of the study hospital as reported annually by the hospital to the Office of Statewide Health Planning and Development.^18^ Readmission was defined as the patient being admitted to the same hospital again within 30 days of his or her last discharge (regardless of whether the readmission was planned or unplanned).

Adverse event(s) represents a binary variable indicating whether at least 1 of 3 types of AEs—drug events, infections, and device-related AEs—occurred during the hospitalization (see eTable 2 in the Supplement for a full list of AEs by category). At the study institution, AEs are identified by medical record coders employed at the hospital who conduct a medical record review for each hospitalization and subsequently determine whether AEs occurred during the patient visit. We separately enlisted the help of a coder at the hospital to further check *International Classification of Diseases, Ninth Revision, Clinical Modification* codes and categorize the AEs in our data, following prior research.^19,20^

### Physician Types and Characteristics

Our sample includes 95 physicians, consisting of 39 hospitalists and 56 general pediatricians. We classified the type of physician (hospitalist vs general pediatrician) based on each physician’s professional affiliations at the hospital provided by the human resources department. Hospitalists were general pediatricians who work solely in the inpatient setting either as full-time equivalents or on a contract basis. Those whose primary affiliation was the hospital medicine division were classified as hospitalists. General pediatricians were hospital-employed physicians who were on the inpatient service approximately 2 weeks per year and otherwise provided care at the hospital’s outpatient clinic. During our study period, hospitalists and general pediatricians were assigned to patients based on age, condition, and bed and nursing availability, a decision directed largely by nursing staff and a designated hospitalist who facilitated coordination of patient assignments. Accordingly, general pediatricians were not the patients’ primary care physicians. Such criteria were intended to be less biased and enable a relatively equal proportion of patients distributed across both groups.

Our analysis included adjustments for physician characteristics obtained from the state’s medical board licensure website and documents that are publicly available online.^14^ We included binary variables for physicians’ sex, part-time service status (<20 hours per week) in the clinical setting, non-US medical school graduate status, and completion of residency training at the study hospital. Based on prior work showing that hospitalists’ years of experience can influence patient outcomes,^15^ we also included a measure of the number of years between each physician’s completion of medical school and each hospitalization, although this measure does not reflect experience specifically in the inpatient context.

### Patient Characteristics and Case-Mix Adjustment

All outcome measures were analyzed in multivariate models adjusted for patient characteristics, including age, sex, insurance type (Medi-Cal, health maintenance organization, Blue Cross, or other), and hospitalization during influenza season (between October and April). As a measure of patient complexity, we used the Chronic Condition Indicator (CCI) system,^21^ which creates binary variables for diagnosis codes from the *International Classification of Diseases, Ninth Revision, Clinical Modification* to identify chronic and nonchronic conditions and categorizes conditions into 18 organ systems, disease categories, or other categories. We adjusted our models for the number of chronic conditions (0, 1, 2, or ≥3) documented for each hospitalization and the types of conditions; 17 of the 18 CCIs were included in the analysis, excluding CCI 11 (“complications of pregnancy, childbirth, and the puerperium”). Based on this algorithm, 16.0% of our sample encounters (228 of 1423) did not have chronic medical conditions, 36.3% (516 of 1423) had a chronic condition with 1 body system, 27.9% (397 of 1423) had chronic conditions in 2 systems, and the remaining 19.8% (282 of 1423) had chronic conditions in 3 or more systems.

### Statistical Analysis

Statistical analysis was performed between July 1, 2017, and October 10, 2018. Descriptive analysis was used to examine patient and physician characteristics, comparing across hospitalists and general pediatricians using either Wilcoxon-Mann-Whitney tests for binary variables and *t* tests for continuous variables. To determine the association between the study outcomes and care provided by a hospitalist or general pediatrician, we used generalized linear models with adjustments for patient characteristics. We analyzed models with and without adjustments for physician characteristics. For continuous variables (LOS and total costs, which were both positively skewed), we used generalized linear models with a gamma distribution and log link. For binary outcomes (readmission rate, AEs, and device-related AEs), we used generalized linear models with a binomial distribution and logit link. Standard errors for these models were clustered at the physician level. However, we were unable to use our preferred specification (generalized linear models with physician-level clustering) for the outcomes of adverse drug events and infections since these models included right-hand variables that predicted perfect failure. Instead, for these 2 outcomes, we fit logistic models by penalizing the maximum likelihood regression with unclustered SEs because clustering is not compatible with this model.^22^ Penalization introduced an acceptable degree of bias into the model as a means to reduce the highly correlated parameter estimates’ variability.^23^ With the exception of models estimating drug events and infections, models had SEs clustered by physician. All models reported odds ratios. *P* values from all models were from 2-sided tests, and results were deemed statistically significant at *P* < .05. For ease of interpretation, all regression coefficients were also converted into predicted probabilities. Analyses were performed in Stata, version 14 (StataCorp).

## Results

### Sample Characteristics

The study consisted of 1423 hospitalizations. Hospitalists cared for 870 patients (61.1%), and general pediatricians cared for 553 patients (38.9%). Among the physicians, there were 57 women and 38 men; physicians were a mean (SD) 11.1 (8.1) years out of medical school. The sample included 697 male patients (49.0%) and 726 female patients (51.0%) with an overall mean (SD) age of 6.1 (6.3) years. Table 1 compares characteristics of patients assigned to a hospitalist vs general pediatrician. There were no significant differences across physician type for patient sex, insurance type, or CCI. However, patients cared for by general pediatricians were younger than those cared for by hospitalists (mean [SD] age, 5.4 [6.0] vs 6.5 [6.4] years; *P* = .001) but had similar mean (SD) Chronic Condition Indicator scores (1.5 [1.0] vs 1.5 [1.0]). Hospitalists also cared for 539 patients (62.0%) hospitalized during flu season, while general pediatricians cared for 300 patients (54.2%) hospitalized during flu season (*P* = .004).

**Table 1.** Comparison of Patient and Physician Characteristics Across Hospitalist and Generalist Attendings

Characteristic	Primary Attending Physician Assigned		*P* Value
	Hospitalist	General Pediatrician	
Total patient encounters, No.	870	553	NA
Patient characteristics			
Age, mean (SD), y^a^	6.5 (6.4)	5.4 (6.0)	.001^b^
Male, No. (%)	428 (49.2)	269 (48.6)	.84^c^
Insurance type, No. (%)			
Medi-Cal	639 (73.4)	425 (76.9)	.14^c^
HMO	94 (10.8)	63 (11.4)	
Blue Cross	115 (13.2)	52 (9.4)	
Other	22 (2.5)	13 (2.4)	
CCI score, mean (SD)	1.5 (1.0)	1.5 (1.0)	.10^b^
Flu season assignment, No. (%)	539 (62.0)	300 (54.2)	.004^c^
Physician characteristics			
Female physician, No./total No. (%)	24/39 (61.5)	33/56 (58.9)	.006^c^
Part-time (<20-h clinical setting), No./total No. (%)	3/39 (7.7)	10/56 (17.9)	<.001^c^
Residency at subject hospital, No./total No. (%)	13/39 (33.3)	38/56 (67.9)	<.001^c^
Foreign medical school, No./total No. (%)	1/39 (2.6)	2/56 (3.6)	<.001^c^
Time since medical school, mean (SD), y	7.9 (3.8)	16.0 (10.3)	<.001^b^

Abbreviations: CCI, Chronic Condition Indicator; HMO, health maintenance organization; NA, not applicable.

^a^ For patients cared for by general pediatricians, ages are 21 years or younger, and for those cared for by hospitalists, ages are 23 years or younger.

^b^ Determined by use of the *t* test.

^c^ Wilcoxon-Mann-Whitney test.

A total of 33 of 56 general pediatricians (58.9%) and 24 of 39 hospitalists (61.5%) were women (*P* = .006), 2 of 56 general pediatricians (3.6%) and 1 of 39 hospitalists (2.6%) had a degree from a foreign medical school (*P* < .001), 10 of 56 general pediatricians (17.9%) and 3 of 39 hospitalists (7.7%) were part-time clinicians (*P* < .001), and 38 of 56 general pediatricians (67.9%) and 13 of 39 hospitalists (33.3%) completed their residency at the study hospital (*P* < .001) (Table 1). General pediatricians also had more experience practicing medicine, having graduated medical school a mean (SD) of 16.0 (10.3) years ago vs 7.9 (3.8) years for hospitalists (*P* < .001).

### Adjusted Differences in Outcomes

In multivariate models adjusting for patient-level features, there were no significant differences between general pediatricians and hospitalists for mean length of stay (4.7 vs 4.6 days), total costs ($14 490 vs $15 200), and estimated 30-day readmission rate (8.9% vs 6.4%), and results were similar with adjustments for physician characteristics. Table 2 presents the adjusted comparisons of patient outcomes for hospitalists vs general pediatricians. The first set of results was adjusted only for patient characteristics, and the second set was adjusted for both patient and physician characteristics. In both models, there was no significant difference in LOS, total costs, or readmission rates when a general pediatrician vs hospitalist was assigned as the primary attending physician. However, in models adjusted for physician characteristics, AEs were significantly less common (odds ratio, 0.48; 95% CI, 0.23-0.97; *P* = .04) under the care of a general pediatrician. This finding reflected a predicted probability of AEs of 2.5% for general pediatricians vs 5.1% for hospitalists.

**Table 2.** Summary Results of Patient Outcomes When Cared for by a General Pediatrician vs a Hospitalist

Outcome	Mean (SD) Value	GLM Without Physician Characteristics^a^^,^^b^				GLM With Physician Characteristics^a^^,^^b^^,^^c^			
		Sample Size, No.	General Pediatrician (vs Hospitalist) as Primary Attending Physician (95% CI)^d^	Probability With General Pediatrician Assigned	Probability With Hospitalist Assigned	Sample Size, No.	General Pediatrician (vs Hospitalist) as Primary Attending (95% CI)^d^	Probability With General Pediatrician Assigned	Probability With Hospitalist Assigned
Length of stay, d	4.7 (8.2)	1423	0.02 (−0.14 to 0.17)	4.7	4.6	1418	−0.03 (−0.21 to 0.15)	4.56	4.7
Total costs, $	14 921 (30 204)	1423	−0.05 (−0.22 to 0.12)^e^	14 490	15 200	1418	−0.11 (−0.30 to 0.07)^e^	13 847	15 513
Readmission rate, %	7.1 (2.6)	1388	1.49 (0.97 to 2.29)	8.9	6.4	1383	1.33 (0.77 to 2.31)	8.4	6.6
Adverse event(s), %^f^	4.1 (2.0)	1423	0.57 (0.32 to 1.01)^g^	2.9	4.9	1418	0.48 (0.23 to 0.97)^h^	2.5	5.1
Adverse drug event, %	1.2 (1.1)	1423	0.79 (0.28 to 2.23)	1.0	1.3	1418	0.54 (0.13 to 2.24)	0.8	1.7
Infection, %	0.6 (7.4)	1040	0.41 (0.08 to 2.27)	0.4	1.0	1418	0.36 (0.06 to 2.26)	0.4	1.4
Device-related adverse event, %	2.2 (14.8)	1388	0.34 (0.12 to 1.00)^i^	1.1	3.0	1383	0.32 (0.09 to 1.14)^g^	1.0	2.8

Abbreviation: GLM, generalized linear model.

^a^ All models clustered by physician and adjusted for patient characteristics including age, sex, insurance type, and Chronic Condition Indicator. However, adverse drug event and infection outcomes were not clustered by physician.

^b^ All models are GLM except for adverse drug event and infection outcomes, which use the *firthlogit* command in Stata.

^c^ Physician characteristics include sex, foreign medical school, years of experience after medical school, part-time status, and whether residency was completed at subject hospital.

^d^ Readmission rate, adverse events, adverse drug event, infection, and device event outcomes are reported as odds ratios. Length of stay and total cost outcomes are reported as coefficients.

^e^ Coefficient reflects the percentage change estimated in the GLM model.

^f^ Excludes surgical events.

^g^ *P* ≤ .10.

^h^ *P* = .042.

^i^ *P* = .049.

The Figure and Table 2 also compare adverse drug events, infections, and device-related AEs as a percentage of hospitalists’ and general pediatricians’ respective total patient assignments. We found no significant differences across groups for adverse drug events and infections. However, device-related AEs were more common among hospitalists (3.0% vs 1.1%; odds ratio, 0.34; 95% CI, 0.12-1.00; *P* = .049). This difference became nonsignificant after adjustment for physician characteristics. When assessing the effect of adding physician characteristics to our model (Table 3), device-related AEs were significantly more common among physicians who completed their residency training at the subject hospital (odds ratio, 1.97; 95% CI, 1.06-3.64; *P* = .03) and those who had fewer years of experience (odds ratio, 0.90 per year; 95% CI, 0.82-0.98; *P* = .01).

**Figure.**
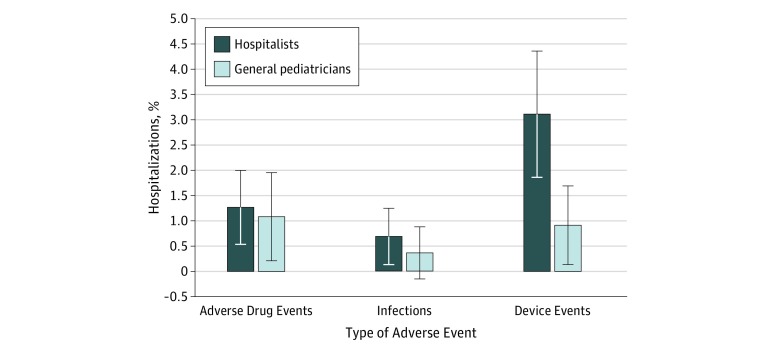
Percentage of *International Classification of Diseases, Ninth Revision, Clinical Modification* Documented Adverse Events Percentage of total adverse events among patient encounters for hospitalists (n = 870) and general pediatricians (n = 553). Error bars indicate 95% CI.

**Table 3.** Patient Outcomes When Cared for by a General Pediatrician vs a Hospitalist, Adjusting for Physician and Patient Characteristics

Characteristic	Length of Stay, % Change (95% CI) (n = 1418)^a^	Total Costs, % Change (95% CI) (n = 1418)^a^	Readmission Rate, OR (95% CI) (n = 1383)^a^	Adverse Event, OR (95% CI) (n = 1418)^a^^,^^b^	Adverse Drug Event, OR (95% CI) (n = 1418)^a^^,^^c^	Infection Adverse Event, OR (95% CI) (n = 1418)^a^^,^^c^	Adverse Device Event, OR (95% CI) (n = 1383)^a^
General pediatrician as primary attending physician	−0.03 (−0.21 to 0.15)	−0.11 (−0.30 to 0.07)	1.33 (0.77 to 2.31)	0.48 (0.23 to 0.97)^d^	0.54 (0.13 to 2.24)	0.36 (0.06 to 2.26)	0.32 (0.09 to 1.14)^e^
Physician characteristics							
Female physician	−0.08 (−0.22 to 0.05)	−0.08 (−0.22 to 0.07)	1.12 (0.75 to 1.66)	1.35 (0.90 to 2.05)	1.59 (0.53 to 4.8)	2.00 (0.45 to 8.75)	1.02 (0.51 to 2.05)
Part-time (<20-h clinical setting)	0.12 (−0.19 to 0.42)	0.14 (−0.17 to 0.45)	0.66 (0.39 to 1.10)	1.09 (0.57 to 2.06)	1.22 (0.29 to 5.14)	1.71 (0.26 to 11.34)	0.79 (0.06 to 10.97)
Residency at subject hospital	−0.02 (−0.17 to 0.14)	−0.01 (−0.18 to 0.15)	1.11 (0.74 to 1.66)	1.11 (0.66 to 1.86)	0.71 (0.23 to 2.14)	1.27 (0.30 to 5.36)	1.97 (1.06 to 3.64)^f^
Foreign medical school	0.06 (−0.30 to 0.42)	−0.01 (−0.33 to 0.31)	0.80 (0.44 to 1.46)	1.33 (0.49 to 3.61)	0.30 (0.02 to 5.76)	0.81 (0.03 to 23.64)	6.56 (0.43 to 100.15)
Time since medical school	0.00 (−0.01 to 0.01)	0.00 (−0.01 to 0.02)	1.01 (0.98 to 1.04)	1.00 (0.96 to 1.04)	1.07 (1.00 to 1.13)	1.04 (0.94 to 1.15)	0.90 (0.82 to 0.98)^g^

Abbreviation: OR, odds ratio.

^a^ Readmission rate, adverse events, adverse drug event, infection, and device-related adverse event outcomes report odds ratios. Length of stay and total cost outcomes report coefficients. All models are clustered by physician and adjusted for patient characteristics including age, sex, insurance type, and Chronic Condition Indicator. However, adverse drug event and infection outcomes were not clustered by physician.

^b^ Excludes surgical events.

^c^ All models are generalized linear models except for adverse drug event and infection outcomes, which use the *firthlogit* command in Stata.

^d^ *P* = .04.

^e^ *P* ≤ .10.

^f^ *P* = .03.

^g^ *P* = .01.

## Discussion

Pediatric hospitalists are increasingly supplanting general pediatricians in providing inpatient care.^9^ The growing trend toward hospitalists is likely to continue for many reasons: one of the foremost being that the specialty has emerged to focus on the complexities of providing care to patients in the inpatient context. Furthermore, general pediatricians are increasingly choosing to relinquish their hospital privileges to focus on their outpatient practices, leaving the role of a generalist physician in the inpatient setting to a hospitalist. In recognition of these trends and as general pediatricians spend more time gaining expertise in outpatient care and less time building skills in the inpatient environment, it is unclear how outcomes differ in care provided by general pediatricians relative to care provided by hospitalists for hospitalized children.^24^ Our study uses nearly 6 years of data from an academic children’s hospital to analyze differences in LOS, total costs, 30-day readmission rate, and the occurrence of AEs when inpatient care is provided to children by pediatric hospitalists vs general pediatricians. We found that most outcome measures did not significantly differ between hospitalists and general pediatricians. Specifically, the results indicate that care delivered by hospitalists and general pediatricians in our sample had similar LOS, total costs, and readmission rates.

We did identify suggestive differences in AEs between hospitalists and general pediatricians. Hospitalist care was associated with significantly more device-related AEs; this effect was attenuated after adjusting for physician characteristics (notably, years of experience practicing medicine after medical school). This finding suggests that, compared with general pediatricians, hospitalists with less time practicing medicine may be at higher risk for device-related AEs. Alternatively, it may be that hospitalists are more likely to treat patients with devices, which we could not measure directly. Another possible explanation is that hospitalists and general pediatricians differed in their documentation using the hospital electronic record system, which could lead to differences in coding frequency for AEs; however, in our study, AE coding was done by an independent record reviewer other than the physician, which should reduce the risk of reporting bias.

In a study examining 44 tertiary care children’s hospitals in the United States, device-related AEs accounted for or complicated 3.3% of inpatient stays and were associated with a mean of more than 17 000 total visits per year in pediatric patients.^14^ Given the prevalence of device-related AEs in children, our research offers insight into factors associated with the characteristics of physicians caring for these patients. More broadly, our study has important implications for hospitals considering transitioning inpatient service entirely to the hospitalist role, which was associated in our data with significantly more device-related AEs. These results also indicate that less experienced physicians may be more prone to device-related AEs. Our findings suggest there are not likely to be any major changes in costs, LOS, or readmissions, but occurrence of AEs—particularly those related to devices—is an area for closer monitoring during such a transition. These issues are also relevant for hospitals that still have general pediatric and hospitalist inpatient services, in which general pediatricians work most of the time in outpatient service and a few weeks per year in inpatient service. Our results suggest that such an approach is unlikely to be harmful to care quality, although this model has potential implications for overall workforce needs, position turnover, and physician job satisfaction, which were beyond the scope of our study and would be important directions for future research.

### Limitations and Future Research

The main limitation of our analysis is that patient assignments to hospitalists and general pediatricians were not random. We adjusted for observable patient and physician characteristics but cannot rule out unmeasured confounding. One notable feature in our sample was that the assigned general pediatrician was not a patient’s primary care physician since, at our study hospital, primary care physicians provided only social visits for their hospitalized patients and were not initially assigned to be responsible for care. However, we also acknowledge that, since the general pediatricians in our sample practice at the study hospital, our findings may not be generalizable to community general pediatricians with hospital admitting privileges.

Some limitations exist in our outcome measures. First, our method for identifying AEs may not capture all relevant events. Categorizing AEs using *International Classification of Diseases, Ninth Revision, Clinical Modification* codes is a conservative measure^25^ that has been used in prior research,^26^ and the fact that these codes were verified by independent medical record reviewers other than the treating physician reduced the likelihood of systematic bias by physician specialty. Second, our cost outcome reflects the practice of applying a year-specific cost-to-charge ratio to the total charges related to the patients’ care, but this method does not account for variation in this ratio across procedures and conditions; however, it is unclear why this method would produce any bias systematically correlated with physician specialty. Third, the 30-day readmission rate reflects both planned and unplanned admissions, as our data set did not enable us to distinguish between them. However, it is likely that most admissions were unplanned because our sample contains only hospitalizations in which a general pediatrician or hospitalist was the primary attending physician; planned admissions in this hospital typically require subspecialists to be the primary attending physician.

Because our data set came from a larger study of generalist and subspecialist pediatricians, our sample excludes many common pediatric conditions, such as asthma and bronchiolitis. Future work should analyze the common conditions missing from this analysis, which could show notable variations in general pediatrician and hospitalist practice. Our sample focuses on a limited number of conditions (57) across 7 subspecialties, which may restrict generalizability, and our sample size may also have lacked power to detect smaller but still clinically relevant differences in care outcomes. However, our sample includes conditions that have similar levels of complexity when we compare CCI scores with other studies, one on a national level^4^ and one in another state (New York).^27^ Furthermore, severity and complexity of conditions may extend beyond the count of chronic conditions we used in our CCI measure. In other words, a patient can have a chronic condition, such as asthma, but in a mild, moderate, or severe form, which our analysis cannot distinguish. The fact that our results were similar in sensitivity analyses without CCIs suggests that this limitation is not driving our findings.

## Conclusions

In a study of more than 1400 hospitalized pediatric patients at a tertiary care medical center, we found similar costs, LOS, and readmission rates for patients cared for by hospitalists compared with those cared for by general pediatricians. However, we found higher rates of AEs—particularly device-related AEs—associated with hospitalist care, suggesting that this is an area for monitoring as the hospitalist model becomes increasingly common in the care of pediatric inpatients.

## Notes

Supplement. **eTable 1.** List of Diagnoses Included in the Sample **eTable 2.** Error Types as Categorized by *ICD-9-CM* Codes and Number of Occurrences in the Sample
