# Incorrect Descriptive Statistical Data

**DOI:** 10.1001/jamanetworkopen.2018.7783

**Published:** 2019-01-25

In the Original Investigation titled “Adverse Events and Patient Outcomes Among Hospitalized Children Cared for by General Pediatricians vs Hospitalists,”^1^ published December 7, 2018, some of the *P* values comparing physician characteristics reflected the full sample (1423 hospitalizations); instead, they should have been calculated off of the ratios of physicians demonstrating those characteristics. In addition, a few of the comparisons have been updated with more appropriate statistical tests (χ^2^ and Fisher exact tests). These modifications to the abstract, the Methods and Results sections, and Table 1 do not change the central findings or the main results of the study. All coefficients for main effects (independent variables) and outcomes remain the same, as do their significances. Only a few of the descriptive statistics related to physician characteristics between hospitalists vs general pediatricians are no longer significant, but these are unadjusted numbers and not part of the more critical findings of the study. This article was corrected online.^1^
