# Supplementary material for: Adverse Events and Patient Outcomes Among Hospitalized Children Cared for by General Pediatricians vs Hospitalists
Source: JAMA Netw Open. 2018 Dec 7;1(8):e185658. doi: 10.1001/jamanetworkopen.2018.5658 (PMC6324330; doi:10.1001/jamanetworkopen.2018.5658)
Supplement: Supplement. — eTable 1. List of Diagnoses Included in the Sample eTable 2. Error Types as Categorized by ICD-9-CM Codes and Number of Occurrences in the Sample [file jamanetwopen-1-e185658-s001.pdf]

Supplementary Online Content

Atkinson MK, Schuster MA, Feng JY, Akinola T, Clark KL, Sommers BD. Adverse events and patient outcomes among hospitalized children cared for by general pediatricians vs hospitalists. *JAMA Netw Open.* 2018;1(8):e185658. doi:10.1001/jamanetworkopen.2018.5658

eTable 1. List of Diagnoses Included in the Sample
eTable 2. Error Types as Categorized by *ICD-9-CM* Codes and Number of Occurrences in the Sample

This supplementary material has been provided by the authors to give readers additional information about their work.

© 2018 Atkinson MK et al. *JAMA Network Open.*

eTable 1. List of Diagnoses Included in the Sample

No.	Diagnosis
1	Acute disseminated encephalomyelitis (ADEM), encephalopathy
2	Guillain-Barre, Chronic inflammatory demyelinating polyneuropathy (CIDP), other demyelinating disorders
3	Infantile spasms
4	Movement disorders, dystonia
5	Opsoclonus-myoclonus
6	Seizure/epilepsy - known for breakthrough or intractable, vs chronic epilepsy in patient admitted for other diagnosis (i.e. pneumonia)
7	Stroke - new work-up, or with unrelated problem
8	Weakness - acute
9	Adrenal insufficiency
10	Congenital adrenal hyperplasia
11	Craniopharyngioma
12	Cushing syndrome
13	Diabetes insipidus
14	Diabetic ketoacidosis
15	Disorder of menstruation & abnormal bleeding from female genital tract
16	Hypercalcemia
17	Hypoaldosteronism
18	Hypoparathyroidism
19	Hypothyroidism
20	Ascites
21	Attention to colostomy, ileostomy, J-tube, G-tube
22	Chronic liver disease
23	Complication due to central venous catheter (CVC)
24	Congenital biliary atresia
25	Conjugated hyperbilirubinemia
26	Constipation, not otherwise specified
27	Constipation, related to chronic GI disease
28	Esophagitis
29	Gastroesophageal reflux
30	Hematochezia
31	Hepatitis, autoimmune
32	Intestinal dysmotility - small bowel
33	Pancreatitis, acute
34	Pancreatitis, chronic

© 2018 Atkinson MK et al. *JAMA Network Open.*

No.	Diagnosis
35	Persistent vomiting
36	Portal hypertension
37	Histiocytic disorders (HLH), admitted for any reason
38	Histiocytic disorders (LCH), admitted for any reason
39	Cystic fibrosis (CF) with admission related to underlying CF (e.g. pneumonia, pancreatitis)
40	Ankylosing spondylitis
41	Arteritis
42	Chronic pain
43	Dermatomyositis
44	Familial mediterranean fever
45	Fibromyalgia
46	Overlap syndrome
47	Pauciarticular jia
48	Systemic sclerosis
49	Wegeners granulomatosis
50	Mechanical complication due to cardiac pacemaker
51	Kawasaki's Disease
52	Endocarditis
53	Cyanosis
54	Cardiomyopathy
55	Myocarditis
56	Pericardial effusion
57	Syncope

© 2018 Atkinson MK et al. *JAMA Network Open.*

eTable 2. Error Types as Categorized by *ICD-9-CM* Codes and Number of Occurrences in the Sample

***ICD-9-CM* Code**	**Code Label**	**Number of occurrences**
**Adverse Drug Event**		
E933.1	ADV EFF ANTINEOPLASTIC	37
284.11	ANTIN CHEMO INDCD PANCYT	13
693	DRUG DERMATITIS NOS	13
E932.0	ADV EFF CORTICOSTEROIDS	10
E934.6	ADV EFF GAMMA GLOBULIN	10
E935.2	ADV EFF OPIATES	8
E930.0	ADV EFF PENICILLINS	7
288.03	DRUG INDUCED NEUTROPENIA	7
995.29	ADV EFF MED/BIOL NEC/NOS	6
E947.8	ADV EFF MEDICINAL NEC	6
285.3	ANEMIA D/T ANTINEO CHEMO	6
357.6	NEUROPATHY DUE TO DRUGS	6
E936.3	ADV EFF ANTCONVL NEC/NOS	5
E938.4	ADV EFF GEN ANES NEC/NOS	5
284.12	OTH DRG INDCD PANCYTOPNA	3
E933.0	ADV EFF ANALLRG/ANTEMET	2
E930.8	ADV EFF ANTIBIOTICS NEC	2
E939.3	ADV EFF ANTIPSYCHOTC NEC	2
E931.7	ADV EFF ANTIVIRAL DRUGS	2
E939.4	ADV EFF BENZODIAZ TRANQ	2
292.12	DRUG PSY DIS W HALLUCIN	2
909.5	LTE EFCT ADVRS EFCT DRUG	2
962	POIS-CORTICOSTEROIDS	2
698.9	PRURITIC DISORDER NOS	2
E858.0	ACC POISON-HORMONES	1
E933.3	ADV EFF ALKALIZING AGENT	1
E932.4	ADV EFF ANT PITUITARY	1
E943.0	ADV EFF ANTACIDS	1
E930.1	ADV EFF ANTIFUNG ANTBIOT	1
E931.9	ADV EFF ANTINFCT NEC/NOS	1
E935.6	ADV EFF ANTIRHEUMATICS	1
E932.8	ADV EFF ANTITHYROID AGNT	1
E945.4	ADV EFF ANTITUSSIVES	1
E931.5	ADV EFF ANTPROTAZOAL NEC	1
E935.4	ADV EFF AROM ANALGSC NEC	1
E948.8	ADV EFF BACT VAC NEC/NOS	1
E937.0	ADV EFF BARBITURATES	1

© 2018 Atkinson MK et al. *JAMA Network Open.*

ICD-9-CM Code	Code Label	Number of occurrences
E939.2	ADV EFF BUTYROPHEN TRANQ	1
E930.5	ADV EFF CEPHALOSPORIN	1
E939.6	ADV EFF HALLUCINOGENS	1
E936.1	ADV EFF HYDANTOIN DERIV	1
995.2	ADV EFF MED/BIOL SUB NOS	1
E932.2	ADV EFF OVARIAN HORMONES	1
E932.5	ADV EFF POST PITUITARY	1
E931.0	ADV EFF SULFONAMIDES	1
E930.4	ADV EFF TETRACYCLINE	1
708	ALLERGIC URTICARIA	1
995.1	ANGIONEUROTIC EDEMA	1
780.39	CONVULSIONS NEC	1
292.81	DRUG-INDUCED DELIRIUM	1
780.66	FEB NONHEMO TRANSF REACT	1
536.49	GASTROSTOMY COMP NEC	1
458.9	HYPOTENSION NOS	1
139.8	LATE EFF INFECT DIS NEC	1
528.01	MUCOSITS D/T ANTINEO RX	1
782.1	NONSPECIF SKIN ERUPT NEC	1
999.59	OTHER SERUM REACTION	1
965.4	POIS-AROM ANALGESICS NEC	1
971.3	POISONING-SYMPATHOLYTICS	1
965.61	POIS-PROPIONIC ACID DERV	1
698.8	PRURITIC CONDITIONS NEC	1
405.99	SECOND HYPERTENSION NEC	1
307.9	SPECIAL SYMPTOM NEC/NOS	1
780.2	SYNCOPE AND COLLAPSE	1
999.89	TRANSFUSION REACTION NEC	1
E980.4	UNDET POIS-MED AGNT NEC	1
**Device Event**		
996.74	COMP-OTH VASC DEV/GRAFT	140
996.01	MALFUNC CARDIAC PACEMAKE	25
996.1	MALFUNC VASC DEVICE/GRAF	17
996.02	MALFUNC PROSTH HRT VALVE	13
E878.2	ABN REACT-ANASTOM/GRAFT	11
E878.1	ABN REACT-ARTIF IMPLANT	10
996.59	MALFUNC OTH DEVICE/GRAFT	7
996.62	REACT-OTH VASC DEV/GRAFT	6
996.57	COMPLCATION-INSULIN PUMP	5

© 2018 Atkinson MK et al. *JAMA Network Open.*

ICD-9-CM Code	Code Label	Number of occurrences
E879.9	ABN REACT-PROCEDURE NOS	4
996.79	COMP-INT PROST DEVIC NEC	3
996.71	COMP-HEART VALVE PROSTH	2
996.72	COMP-OTH CARDIAC DEVICE	2
536.42	GASTROSTOMY COMP - MECH	2
996.69	REACT-INT PROS DEVIC NEC	2
996.67	REACT-OTH INT ORTHO DEV	2
997.4	SURG COMP-DIGESTV SYSTEM	2
E879.0	ABN REACT-CARDIAC CATH	1
E879.2	ABN REACT-RADIOTHERAPY	1
E879.3	ABN REACT-SHOCK THERAPY	1
426	ATRIOVENT BLOCK COMPLETE	1
996.75	COMP-NERV SYS DEV/GRAFT	1
996.56	COMP-PERITON DIALYS CATH	1
780.6	FEVER NOS	1
998.11	HEMORRHAGE COMPLIC PROC	1
E874.8	INSTRMNT FAIL-PROCED NEC	1
E874.1	INSTRUMNT FAIL-INFUSION	1
996.09	MALFUNC CARD DEV/GRF NEC	1
996.52	OTH TISSUE GRAFT MALFUNC	1
**Infection**		
998.59	OTHER POSTOP INFECTION	11
536.41	GASTROSTOMY INFECTION	10
999.33	LCL INF DT CEN VEN CTH	3
999.32	BLOOD INF DT CEN VEN CTH	2
998.51	INFECTED POSTOP SEROMA	1

© 2018 Atkinson MK et al. *JAMA Network Open.*
